# Integrated Management Strategies Increase Cottonseed, Oil and Protein Production: The Key Role of Carbohydrate Metabolism

**DOI:** 10.3389/fpls.2017.00048

**Published:** 2017-01-30

**Authors:** Hongkun Yang, Xinyue Zhang, Binglin Chen, Yali Meng, Youhua Wang, Wenqing Zhao, Zhiguo Zhou

**Affiliations:** Key Laboratory of Crop Physiology & Ecology, Jiangsu Collaborative Innovation Center for Modern Crop Production, Ministry of Agriculture, Nanjing Agricultural UniversityNanjing, China

**Keywords:** cottonseed, integrated management strategies, seed weight, embryo oil and protein, carbohydrate metabolism

## Abstract

Cottonseed, oil, and protein, as the by-products of cotton production, have the potential to provide commodities to meet the increasing demand of renewable bio-fuels and ruminant feed. An increase in crop yield per unit area requires high-yielding cultivar management with an economic nitrogen (N) rate, an optimal N application schedule, high-yielding plant populations and strong seedlings. Whether the integration of these agronomic practices into a coherent management system can increase the productivity of cotton fiber, embryo oil and protein requires experimental elucidation. In this 2-year study, conventional management practices (CM) were used as a control, and two integrated management strategies (IMS_1_ and IMS_2_) were considered at two soil fertility levels (high soil fertility and low soil fertility) to analyze the metabolic and biochemical traits of cotton embryos. The results illustrate that the cottonseed, oil, and protein yields for IMS_1_ and IMS_2_ were significantly higher than those under CM at both soil fertility levels and the fiber yield increased as well. The IMS regulated the maternal photo thermal environment by delaying the flowering date, resulting in increases in the seed weight. In developing cotton embryos, the IMS increased the embryo weight accumulation rate and biomass partitioning into oil and protein, which were associated with high activities of H^+^-ATPase, H^+^-PPase, sucrose synthase (SuSy), and cell wall invertase (C-INV) and low activities of sucrose phosphate synthase (SPS) and vacuole invertase (V-INV). Increased hexoses (D-fructose, D-glucose) content contributed to the oil and protein contents. These results suggest that increased sucrose/H^+^ symport, sucrose hydrolysis, hexoses synthesis, and cumulative photo-thermal product (PTP), especially in the early stage of embryo growth, play a dominant role in the high productivity of cotton oil and protein.

## Introduction

Cottonseed, as a by-product of cotton production, is the fifth largest oil seed crop, followed by soybean, rapeseed, peanut, and sunflower. Oil and protein are the main storage reserves in cottonseed embryos, and they provide commodities for renewable bio-fuels and ruminant feed (Meyer et al., 2001; O'Brien et al., 2002; Nabi et al., 2009). Increasingly growing demand for these by-products has drawn attention to genome editing (Liu et al., 2000, 2002), cotton breeding (Yu et al., 2012), sustainable cropping systems (Du et al., 2015), and optimal agronomic strategies (Sawan et al., 1988, 2001, 2007) to obtain a high yield and optimal quality in cottonseed. However, stagnant yields (FAO database) with a lower nitrogen (N) use efficiency (Raun and Johnson, 1999; Zhu and Chen, 2002; Foley et al., 2011; Mueller et al., 2012) are a serious problem worldwide. An economic N rate, optimal N management practices, high-yielding plant populations, and strong seedlings are essential for achieving the maximum attainable yield under a given set of environmental conditions.

Optimal nutritional management (Wullschleger and Oosterhuis, 1990b; Bondada et al., 1996; Oosterhuis, 2001; Sapkota et al., 2014), water management (Falkenberg et al., 2007), high-yielding plant populations (Dong et al., 2005, 2010, 2012; Dai et al., 2015), and plant growth regulators (Sawan et al., 1993, 2006; Mao et al., 2014) positively affect leaf photosynthesis, biomass accumulation and crop yields. Recent, studies have suggested that N fertilizer can be used much more efficiently at a moderately lower application rate without any loss in yield (Rochester et al., 2009). An adequate increase in plant density can compensate for a low yield per plant, which is affected by a low N application rate (Dong et al., 2010, 2012; Testa et al., 2016). Furthermore, integrated N management strategies increase crop yield and N efficiency in rapeseed (Weisler et al., 2001), groundnut (Prasad et al., 2002), and tomato (Javaria and Khan, 2011). Additive effects may exist among these agronomic practices, and the integration of these agronomic practices into a coherent management system aimed at obtaining high cottonseed yields and maximum oil and protein output requires further study.

It is not clear why agronomic practices have positive effects on cotton oil and protein. An analysis of the possible mechanism by which integrated management strategies (IMS) promote cottonseed, oil and protein yields requires an examination of embryo weight accumulation and key metabolic events occurring in developing cotton embryos. The embryo maturation stage is characterized by the rapid accumulation of oil and storage protein; Thus, the embryo weight increase rapidly during this stage (Forman and Jensen, 1965). This stage is only 20 days long, but it contributes to 75–80% of the embryo dry weight accumulation in the form of oil and storage protein (Chen et al., 2015). Additionally, numerous studies have suggested the potential contributions of carbon production and metabolism to oil and storage protein in cottonseed (Wullschleger and Oosterhuis, 1990a; Ruan et al., 1997) maize (Braun et al., 2014), and rapeseed (Hua et al., 2014) because oil and protein are ultimately derived from carbon (primarily in the form of sucrose) and are delivered to embryos via transfer cells in seed coats (Murphy et al., 1993; McDonald et al., 1996). However, the early stage of embryo growth corresponds to the most active phase of carbon metabolism (Ruan et al., 2008) and not to the most active phase of seed storage reserves accumulation becoming highly convoluted.

Sucrose is a metabolite and signaling molecule (Wind et al., 2010; Braun et al., 2014), and its transport into sink tissues is energy dependent and thus depends on the activity of H^+^-symporter carrier proteins localized in the plasma membrane (Sauer, 2007). Sucrose uptake and hexoses transport are mediated by sucrose (Baud et al., 2005) and monosaccharide transporters (Sherson et al., 2003) in the plasma membrane. Proton-translocating H^+^-ATPase pumps protons out of cells and acts as the primary source of energy for sucrose/H^+^ symport (Ayre, 2011). It also uses an inorganic pyrophosphate (PPi) gradient, which is driven by H^+^-PPase as its energy source for the transportation of organic solutes, such as sucrose, glucose, fructose, and amino acids (Martinoia et al., 2007). The hydrolysis of sucrose into hexoses is mediated by sucrose synthase (EC 2.4.1.13, SuSy) and cell wall invertase (EC 3.2.1.26 C-INV) (Miller and Chourey, 1992; Koch, 2004). INVs also regulate carbon partitioning in developing seeds (Hendrix, 1990; Ruan and Chourey, 1998). Sucrose phosphate synthase (EC 2.3.1.14, SPS) regulates sucrose synthesis and triggers seed development (Weber et al., 1996; Sturm and Tang, 1999). In addition, hexoses provide carbon skeletons for oil and protein biosynthesis in the embryo and regulate many processes associated with seed growth (Ruan et al., 2008). Based on these studies, carbohydrate metabolism may contribute to the productivity of cotton embryo oil and storage protein. Whether high yields of cottonseed, oil, and protein can be gained through IMS requires investigation of the metabolic levels of sucrose/H^+^ symport, sucrose hydrolysis, and hexoses synthesis.

In this study, an economic N rate, an optimal N application schedule, adequate plant population and seedlings raising methods for cotton production were integrated as a coherent management system. Increases in cottonseed, oil, and protein yields were evaluated in compared with those achieved under conventional management practices to determine the beneficial effects of the IMS on the productivity of cotton embryo oil and protein. To determine if and how the IMS increase cottonseed, oil, and protein yields, field experiments were conducted under the hypothesis that carbohydrate metabolism is an important factor that controls the productivity of cotton embryo oil and storage protein. The cottonseed growth response to the environmental conditions and IMS were quantified. These beneficial effects will help determine the optimal management strategy for improving the yield, quality and commercial value of cotton fiber and cottonseed.

## Materials and methods

### Growth conditions and integrated management strategies

**Figure 1:**
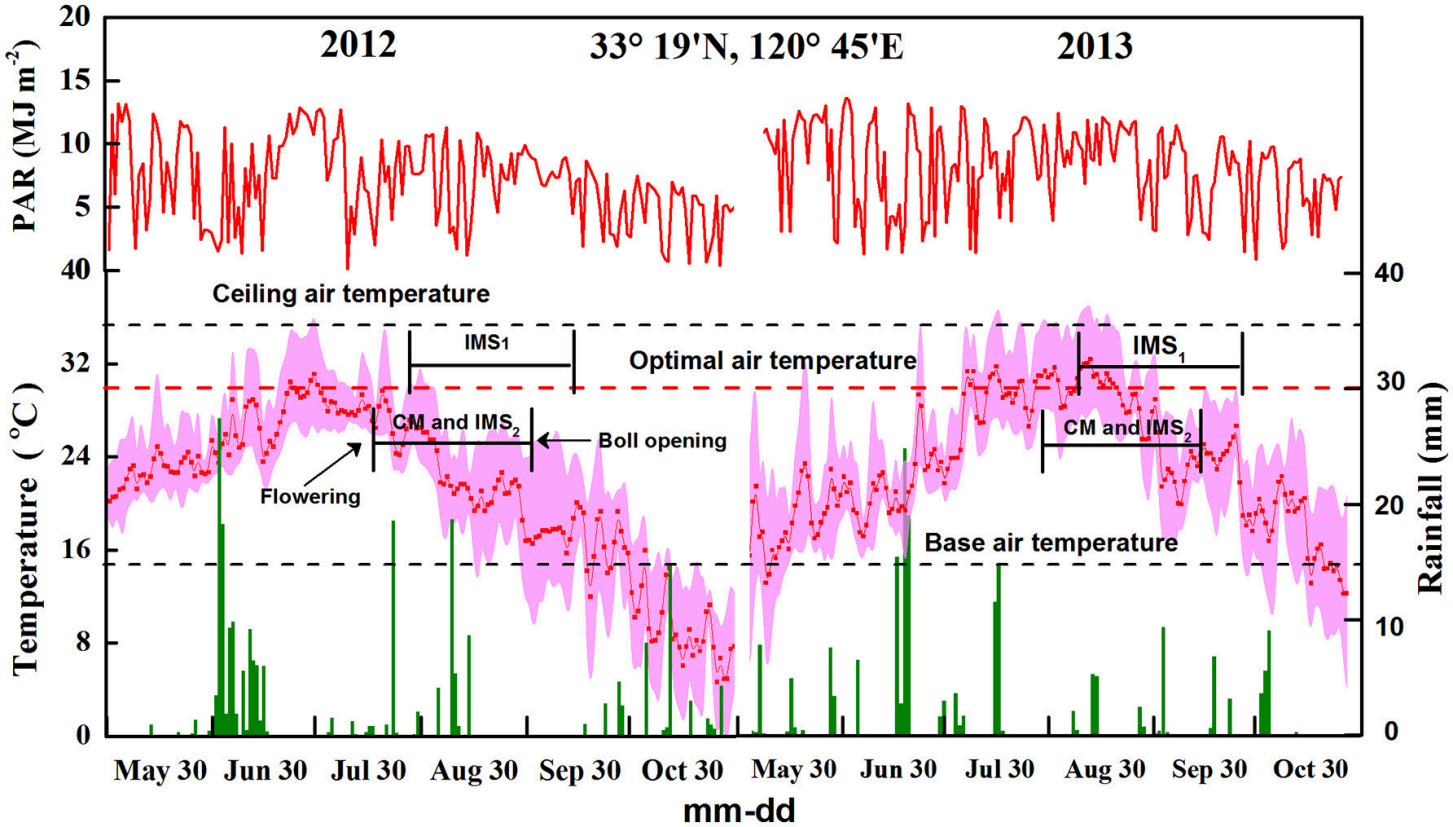
**Photosynthetic active radiation, rainfall, and maximum, minimum, and average temperatures at the experimental site in 2012 and 2013**. A factor of 0.5 was applied for the conversion of solar radiation to photosynthetic active radiation (PAR). The dotted line represents the optimal air temperature for cotton growth.

A widely grown cotton (*Gossypium hirsutum* L.) cultivar called Siza-3 was grown at the Dafeng experimental station (33 19′N, 120 45′E) in Jiangsu, China in 2012 and 2013. Daily meteorological data (solar radiation, air temperature, rainfall) were collected using a weather station (Campbell AG800, Genetics, USA) (Figure 1). The soil at the experimental site was a sandy loam deficient in N and rich in P and K. Two soil fertility levels (FLs; at site 200 meters apart) were identified based on the soil nutrient status (Yang et al., 2016). The soil organic matter and total N contents were significantly different (*P* < 0.05), with the low-fertility soil averaging 14.31 ± 0.62 g kg^−1^ soil organic matter and 0.79 ± 0.04 g kg^−1^ total nitrogen and the high-fertility soil averaging 17.76 ± 0.69 g kg^−1^ soil organic matter and 0.86 ± 0.03 g kg^−1^ total N content, both at a soil depth of 0–20 cm.

Under each soil fertility level, the conventional management practices (CM) and two integrated management strategies (IMS_1_ and IMS_2_) were arranged in a randomized complete block design with three replicates. Each plot was 22 m long and 10 m wide. CM refers to the management practices that are widely used in the Yangtze River cotton-producing region. N was applied at a rate of 300 kg ha^−1^, with 40% applied as basal N fertilizer and the remaining 60% applied at the initial flowering stage. In addition, the plant density of 18,000 plants ha^−1^ was used. Cottonseeds were planted in a nursery bed on April 15, and seedlings were transplanted to the field on May 15 when they had 3–4 true leaves.

IMS_1_ and IMS_2_ were designed according to previously reported management systems for irrigated rice (Peng et al., 2006; Islam et al., 2014) and rapeseed (Rathke et al., 2006). These IMS differed from the CM in terms of N rate, N application schedule, plant density, and seedling-raising methods. IMS_1_ consisted of an economic N application rate (375 kg N ha^−1^), a high plant density (30,000 plants ha^−1^) and the substrate seedling-raising method. For this method, seeds were planted with a bio-organic fertilizer (20 million g^−1^ efficacious living cells and 43.6% organic matter, purchased from Jiangsu Tianniang Ltd., China) at a rate of 10 g per seedling, and the seedlings were mechanically transplanted to the field with substrate on May 15 when they had 3–4 true leaves. The economic N application rate was calculated according to a previously described algorithm (Baker and Young, 2004); 20% was applied as base fertilizer, 25% at the initial flowering stage, 40% at the full blooming stage, and 15% at the end of the flowering stage. Under IMS_2_, the N application rate increased to 525 kg ha^−1^, and the proportional distribution described for IMS_1_ was used. Furthermore, the plant density increased to 30,000 plants ha^−1^ using the seedling transplantation method. The target yield fertilization rate was calculated as previously described (Katayama et al., 1999). In brief, IMS_2_ combined the target yield fertilization rate, high plant density, and seedling transplantation with a target cottonseed yield of 6400 kg ha^−1^.

Notably, the integration of the economic N application rate, N application schedule, optimal plant density, and substrate seedling-raising method were based on previous single-factor experiments (Chen et al., 2015, 2016; Meng et al., 2016), with the expectation that their combined beneficial effect was much stronger than the effect of each factor alone. Additionally, N, P, and K were applied as urea (46% N), ordinary superphosphate (12% P_2_O_5_ and 12% sulfur) and potassium sulfate (50% K_2_O and 18% sulfur), respectivity, at 1.0:0.6:1.1. The N application schedules of IMS_1_ and IMS_2_ were designed to ensure an adequate N supply during the flowering and boll-forming stages. The plant density was adjusted by altering the spacing between rows to facilitate mechanical harvesting and to increase light interception. The primary goals of using the substrate seedling-raising method of IMS_1_ were reduced labor cost, suitability for mechanized transplantation, and strong seedlings. Integrated pest management and furrow irrigation were used to prevent biotic and abiotic stress during the cotton growth period.

### Cottonseed, oil and protein yields

At harvest, the cottonseed yield was determined from the boll number per hectare, seed number per boll, and seed weight. The boll number per hectare was measured by hand picking all open bolls in a 5 m × 5 m sampling area in each plot. The seed number per boll and seed weight were determined from a 50 random samples of cotton bolls representing of each plot. After being de-hulled, the embryos from each plot were analyzed to determine the oil and protein contents. The oil content was determined using the Soxtec method (Avanti 2050 Automatic System) with petroleum ether (30–60°C) as a solvent (Razal et al., 2012). The protein content was quantified using the standard Kjeldahl procedure with a continuous flow analyzer (Bran and Luebbe TRAACS Model 2000 Analyzer). A factor of 6.25 was applied to convert N content to protein content (Wolf et al., 1982). The oil and protein yields were calculated from the cottonseed yield, the ratio of embryo weight to seed weight, and the embryo oil and protein contents.

### Sampling

White flowers at the first node of the 7th–8th sympodial branches were labeled using plastic tags listing the flowering date, and bolls were collected once every 7 days from 10 days after anthesis (DAA) until boll opening at 9:00–10:00 A.M. (local time). The collected bolls were stored at 4°C and quickly delinted using pre-cooled forceps. After their seed lengths and widths (*n* = 20) were determined, the ovules (≥17DAA) were separated into seed coats and embryos. Approximately half of the embryo samples were immersed in liquid nitrogen and stored at −80°C until the enzymatic assays were conducted. The remaining embryos and seed coats were dried at 105°C for 30 min, and then at 60°C to a constant weight to determine the 100-embryo weight and 100-seed weight. The dried embryo samples were ground and passed through a 1-mm sieve and then used to determine the embryo oil and protein contents as well as the starch, sucrose, D-glucose, and D-fructose contents.

### Sucrose, D-glucose, D-fructose, and starch contents

The sucrose, D-glucose, D-fructose, and starch were extracted and quantified according to Hendrix (1993). In brief, the dried and powdered embryos (0.1 g) were extracted three times with 80% ethanol in a water bath at 80°C for 30 min. The ethanol-soluble carbohydrate extract was diluted to a final volume of 25 mL and stored at −20°C to measure the sucrose and hexoses (D-glucose and D-fructose) contents. Assays were performed in a 96-well polystyrene plate, read using a Benchmark microplate reader (Bio-Rad), and were quantified using standard materials.

The ethanol-insoluble residue was used to extract starch (Tang et al., 2014). Dried residue was released in a boiling water bath in 2 mL of distilled water for 15 min and then hydrolyzed with 9.2 M HClO_4_ (2 mL) for 15 min. After this, 4 mL of distilled water was added to the residue and each sample was centrifuged at 4000 rpm for 10 min and extracted again using 2 mL of 4.6 M HClO_4_. The supernatants were combined, and distilled water was added to a final volume of 25 mL. The starch was measured spectrophotometrically at 620 nm using an anthrone reagent, and D-glucose was used as the standard.

## Enzymatic assays

### Plasma membrane H^+^-ATPase and H^+^-PPase activities

#### Plasma membrane extraction

Plasma membrane H^+^-ATPase and H^+^-PPase were extracted, and their activities were determined as previously described with modifications (Bennett et al., 1985; Rocha and De, 1998). The embryos were homogenized in 5 mL of buffer containing 50 mM HEPES-Tris (pH 7.0), 300 mM sucrose, 8 mM EDTA, 2 mM PMSF, 4 mM DTT, and 5% (v/v) glycerol. After adding 2 mL of ether and centrifuging at 12,000 × g for 20 min at 4°C, the supernatant of the homogenate (below the ether layer) was re-centrifuged at 100,000 × g for 10 min at 4°C. Microsomal fractions were suspended in a buffering medium containing 50 mM HEPES-Tris (pH 7.0), 8 mM EDTA and 4 mM DTT. The supernatant homogenates were assayed immediately.

#### H^+^-ATPase assay (EC 3.6.1.35)

The reaction was performed in a volume of 0.5 mL containing 3 mM MgSO_4_, 50 mM, KNO_3_, 50 mM, KCl, 3 mM NaN_3_, 0.125 mM ammonium molybdate and 50 μL of microsomes. After the addition of 50 μL of 20 mM Tris-ATP (which was prepared by adding Na-ATP to a cation exchange resin under acidic conditions, followed by the addition of Tris-HCl to reach pH reached 7), the reaction mixture was incubated at 37°C for 30 min. The inorganic phosphate (Pi) content was evaluated as previously described (Ames, 1966). The H^+^-ATPase activity was expressed as μM Pi g^−1^ fresh weight·h^−1^.

#### H^+^-PPase assay (EC 3.6.1.1)

The standard assay mixture (0.5 mL) contained 30 mM Tris-HCl (pH 6.5), 3 mM MgSO_4_, 50 mM KNO_3_, 0.5 mM NaN_3_, and 0.125 mM ammonium molybdate. After the addition of 50 μL of microsomes to the mixture, the reaction was initiated by adding 50 μL of 20 mM Tris-PPi at 37°C for 20 min and terminated by adding 0.1 mL of 1 M NaOH. The Pi content was measured as previously described (Ames, 1966). The activity of H^+^-PPase is expressed as μM Pi g^−1^ fresh weight·h^−1^.

### Sucrose synthase, invertase, and sucrose phosphate synthase

Enzymes associated with sucrose metabolism were extracted as previously described (King et al., 1997) with modifications. Fresh embryos (0.3 g) were homogenized in 5 mL of a buffer solution containing 50 mM HEPES-NaOH (pH 7.5), 10 mM MgCl_2_, 1 mM Na-EDTA, 1 mM Na-EGTA, 5% (v/v) glycerol, 0.1% (v/v) Triton X-100, 2.5 mM DTT, and 2% (w/v) PVP. After adding 2 mL of ether and centrifuging at 15,000 × g for 20 min at 4°C, the supernatant of the homogenate (below the ether layer) was maintained at 4°C and assayed immediately.

#### Sucrose synthase assays (cleavage direction, SuSy, EC 2.4.1.13)

SuSy activity was measured according to a previously described method (Shu et al., 2009). The reaction mixture contained 20 mM PIPES-KOH buffer (pH 6.5), 100 mM sucrose, 2 mM UDP, and 200 μL of enzyme extract in a total volume of 650 μL. The mixture was incubated in a water bath at 30°C for 30 min and the reaction was subsequently terminated by adding of 250 μL of 500 mM Tricine-KOH buffer (pH 8.3). The SuSy activity was determined according to the amount of fructose produced from sucrose and is expressed as mg fructose g^−1^ fresh weight·h^−1^.

#### Cell wall invertase (C-INV, EC3.2.1.26) and vacuole invertase (V-INV, EC3.2.1.26) assays (Hanft and Jones, 1986)

The reaction mixture contained 0.2 M citrate buffer (pH 4.8), 100 μL of extracted enzyme and 200 μL of 1 M sucrose in a total volume of 2.5 mL. The mixture was incubated in a water bath at 30°C for 30 min, and the reaction was terminated by adding 250 μL of 0.5 M Tricine-KOH. The glucose produced from C-INV was quantified using a previously described method (Nelson, 1944). The data are expressed as mg glucose g^−1^ fresh weight·h^−1^. The method used for the V-INV measurements was similar to that used for the C-INV measurements, except that it was performed in 200 mM citrate buffer (pH 8.0).

#### Sucrose phosphate synthase (SPS, EC 2.4. 1.14) assays

The reaction mixture for the SP (EC 2.4.1.14) assay contained 14 mM uridine diphosphate glucose (UDPG), 50 mM fructose-6-phosphate, and 200 μL of extracted enzyme in a total volume of 650 μL. The reaction mixture was incubated at 30°C for 30 min, and the reaction was terminated by adding 0.1 mL of 1 M NaOH. After the mixture was cooled, the sucrose production was measured at 620 nm. The data are expressed as mg sucrose g^−1^ fresh weight·h^−1^.

### Statistical analyses

A three-way analysis of variance was performed using at least three replicates to examine the effects of the year (Y), soil fertility level (FL), integrated management strategies (IMS), and their interactions on cottonseed, oil and protein yields. These measurements are expressed as the mean ± SE. Fisher's least significant difference (LSD) test was used for statistical analysis. The data were statistically evaluated using SAS software version 9.4. Statistical significance was indicated by *P* < 0.05 or *P* < 0.01. The graphs were plotted using Origin software, version 9.0. Bayesian network-based relationships were analyzed using BayesiaLab software, version 5.4.

The geometry of cottonseeds was estimated as a cone and a half sphere. The seed size was estimated using the following formula:

$$V=\frac{1}{3}\pi {\left(\frac{W}{2}\right)}^{2}L+\frac{4}{3}\pi {\left(\frac{W}{2}\right)}^{3}$$

where V is the seed size, W is the average seed width and L is the average seed length.

The cumulative photo-thermal product (PTP) was calculated using previously described equations (Li et al., 2009) as follows:

(1) $$PTP=\sum PTP_{i}$$

(2) $$PTP_{i}=PAR_{i}\times RTE_{i}$$

(3) $$\begin{array}{l} RTE_{i}=0.5\times \text{RTE}({\text{T}}_{\text{avg}})\;+0.25\times \text{RTE}({\text{T}}_{\text{max}})+0.25\\ \;\times \text{RTE}({\text{T}}_{\text{min}}) \end{array}$$

(4) $$RTE(T)\text{​ }=\;\{\begin{array}{cc} 0 & T\;>\;T_{c},\;or,\;T\;<\;T_{b}\\ {\left(\frac{T-T_{b}}{T_{O}-T_{b}}\right)}^{1+\frac{T_{O}-T}{T_{O}-T_{b}}}\times {\left(\frac{Tc-T}{Tc-T_{b}}\right)}^{\frac{Tc-T_{O}}{Tc-T_{b}}} & T_{b}\leq T\leq T_{O}\\ (\frac{T-T_{b}}{T_{O}-T_{b}})\times {\left(\frac{Tc-T}{Tc-T_{O}}\right)}^{\frac{Tc-T_{O}}{Tc-T_{b}}} & T_{O}\leq T\leq T_{c} \end{array}$$

where RTE is the daily thermal effectiveness. A factor of 0.5 was applied to convert solar radiation to photosynthetically active radiation (PAR). T_avg_, T_max_, and T_min_ represent the daily average, maximum, and minimum air temperatures, respectively. In addition, T_b_, T_O_, and T_c_ represent the base, optimal and ceiling air temperatures for seed development, respectively, which were defined as 15, 30, and 35°C (Chen et al., 2006; Li et al., 2009).

## Results

### Cottonseed, oil and protein yields

Across 2 years, the averaged cottonseed yield under CM was 2410 kg ha^−1^ and 2848 kg ha^−1^ at low and high soil fertility levels, respectively. The cottonseed yield under IMS_1_ and IMS_2_ increased by 22.65 and 32.10% at the low soil fertility level, and by 7.31 and 25.20% at the high soil fertility level, respectively, compared with that under CM (Table 1). The cotton grown at the high soil fertility level using IMS_2_ produced the highest cottonseed yield (3570 kg ha^−1^) and the highest fiber yield (2736 kg ha^−1^). The cottonseed yield increased as the N application rate and plant density increased. Increasing the N application at the flowering and boll-forming stages and using the substrate seedling-raising method were both beneficial to cottonseed yield.

**Table 1:** **Mean cottonseed yields (kg ha^**−1**^) under recommended integrated management strategies (IMS) compared with conventional management practices (CM) at two soil fertility levels in 2012 and 2013**.

**Year**	**Soil fertility levels**	**Integrated management strategies**	**N rates (kg ha^−1^)**	**N application schedule**	**Plant density (plant ha^−1^)**	**Seedling raising method**	**Cottonseed yield (kg ha^−1^)**	**Increase of IMS over CM (%)**	***P***
2012	LF	CM	300	120-180-0-0	18,000	ST	2274		
		IMS_1_	375	75-94-150-56	30,000	SR	2873	26.34	<0.01
		IMS_2_	525	105-131-210-79	30,000	ST	3325	31.60	<0.01
	HF	CM	300	120-180-0-0	18,000	ST	2786		
		IMS_1_	375	75-94-150-56	30,000	SR	3155	13.23	<0.05
		IMS_2_	525	105-131-210-79	30,000	ST	3570	21.95	<0.01
2013	LF	CM	300	120-180-0-0	18,000	ST	2547		
		IMS_1_	375	75-94-150-56	30,000	SR	3031	19.00	<0.01
		IMS_2_	525	105-131-210-79	30,000	ST	3006	15.26	<0.05
	HF	CM	300	120-180-0-0	18,000	ST	2910		
		IMS_1_	375	75-94-150-56	30,000	SR	2950	1.40	ns
		IMS_2_	525	105-131-210-79	30,000	ST	3558	18.22	<0.05

*LF and HF represent the low and high soil fertility levels, respectively. CM and IMS represent conventional management practices and integrated management strategies, respectively. The N application schedules of IMS_1_ and IMS_2_ were timed to coincide with growth-driven N demand by cotton plants (see Materials and Methods). ST: Seeds were planted in a nursery bed, and then transplanted into the field on May 15th. SR: Seeds were planted with bio-organic fertilizer, and seedlings were transplanted to the field with substrate on May 15. The probability (P) of significant treatment difference was determined according to a paired “t” test; ns = not significantly different at P = 0.05*.

**Figure 2:**
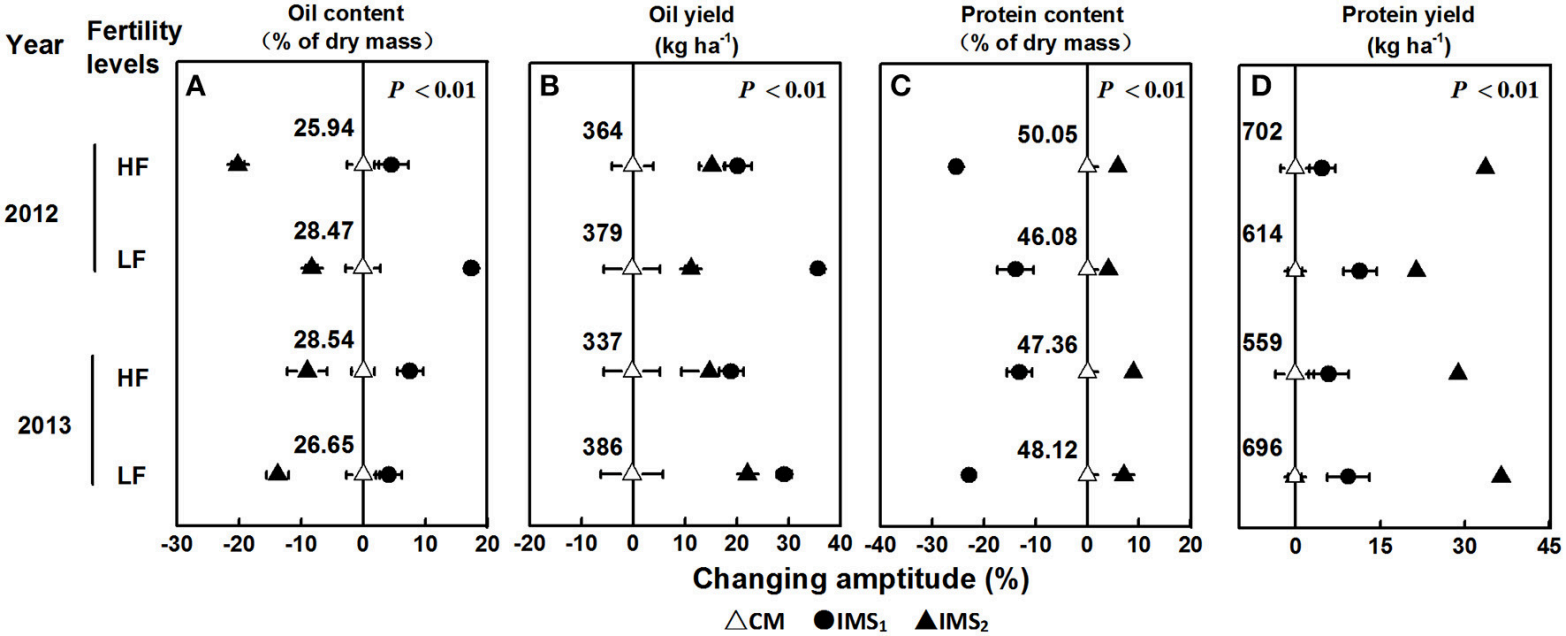
**Changing amplitude (%) of the oil content (A)**, oil yield **(B)**, protein content **(C)**, and protein yield **(D)** in IMS_1_ and IMS_2_ compared to those under CM. CM and IMS represent conventional management practices and integrated management strategies, respectively. The change in amplitude was calculated as follows: Changing amplitude (%) = [IMS–CM] × 100%/IMS. For each sample point, we tested at least 3 independent samples. The data points are the averages, and the bars = SEs.

The oil and protein contents accounted for 75.30% of the total embryo dry weight, and the oil content was significantly negatively correlated with the protein content (*P* < 0.01, Table 2). Averaged over 2 years, the oil content under CM, IMS_1_, and IMS_2_ was 27.40, 30.12, and 24.29%, and the protein content was 47.90, 45.89, and 52.11%, respectively. The oil contents under IMS_1_ markedly increased by 6.49 and 12.71% at low and high soil fertility levels, respectively, compared with those for CM, but the protein content decreased slightly by 2.46 and 5.74% (Figure 2). The protein content under IMS_2_ increased significantly by 5.96 and 11.80% at the low and high soil fertility levels, and the oil content decreased by 9.90 and 12.51%, respectively, compared with that under CM. Furthermore, the increase in the cottonseed yield per 100 kg was accompanied by an increase in the protein content of 0.53% on average, and a decrease in the oil content of 0.45% on average.

**Table 2:** **Embryo oil and protein yields under conventional management practices and two integrated management strategies at two different fertility levels in 2012 and 2013**.

**Soil fertility levels**	**Integrated management strategies**	**Protein content (% of embryo dry mass)**		**Protein yield (kg ha**^**−1**^**)**		**Oil content (% of embryo dry mass)**		**Oil yield (kg ha**^**−1**^**)**	
		**2012**	**2013**	**2012**	**2013**	**2012**	**2013**	**2012**	**2013**
Low soil fertility	CM	46.08b	48.12b	614c	696c	28.47b	26.65b	379c	386c
	IMS_1_	44.97c	46.91c	717b	774b	29.84a	28.84a	476a	476a
	IMS_2_	52.46a	52.81a	992a	978a	23.69c	24.46c	448b	453b
High soil fertility	CM	50.05b	47.36b	702c	559c	25.94b	28.54b	364c	337c
	IMS_1_	44.66c	47.02c	804b	752b	31.42a	29.77a	566a	476a
	IMS_2_	52.21a	50.96a	893a	879a	23.95c	25.08c	410b	433b

*Different letters indicate a significant difference between treatments within the same column during the same year according to the least significant difference (LSD) test (P < 0.05). The treatment was the integrated management of N, plant density, and seedling-raising methods (see Material and Methods). The change in amplitude of the oil content, oil yield, protein content, and protein yield under IMS_1_ and IMS_2_ compared to that under CM is shown in Figure 2*.

IMS_1_ and IMS_2_ showed significant advantages over CM in terms of oil and protein yields (Figure 2). The highest oil yield (566 kg ha^−1^) was produced with IMS_1_, and the highest protein yield (978 kg ha^−1^) was produced with IMS_2_. The oil and protein yields under IMS_1_ increased by 51.04 and 17.78% at the low soil fertility level and by 42.23 and 20.56% at the high soil fertility level, respectively. In addition, the oil and protein yields under IMS_2_ were increased by 13.99 and 24.45% at the low soil fertility level and by 24.43 and 48.37% at the high soil fertility level, respectively. These results indicate that increased cottonseed yield due to an economic N rate, an optimal N application schedule, high-yielding plant populations and the substrate-seedling raising method can compensate for a lower embryo oil content, resulting in greater oil and protein yields.

Significant FL × IMS and Y × FL × IMS interactions were observed for the cottonseed, oil and protein yields (*P* < 0.01) (Table 3). Additionally, significant differences between the 2 years were observed in oil and protein yields but not in the cottonseed yield or protein and oil contents. These results indicate that the IMS promote cottonseed, oil, and protein yields at low and high soil fertility levels, despite the negative correlation between embryo oil and protein contents. The design of management strategies should consider both environmental conditions and soil nutrition status.

**Table 3:** **Results of ANOVA on the effects of year (Y), soil fertility level (FL), integrated management strategies (IMS) and their interactions on the seed yield, seed traits, embryo oil and protein yields in 2012 and 2013**.

**Effect**	***df***	**Seed yield**	**Seed weight**	**Seed size**	**Embryo weight**	**Protein content**	**Protein yield**	**Oil content**	**Oil yield**
Y	1	ns	229.03^**^	169.84^**^	101.02^**^	ns	8.82^**^	ns	9.33^**^
FL	1	84.77^**^	25.61^**^	15.25^**^	83.51^**^	ns	13.62^**^	ns	4.86^*^
IMS	2	380.02^**^	18.96^**^	198.65^**^	24.64^**^	838.89^**^	655.46^**^	237.41^**^	130.52^**^
Y × FL	1	ns	80.52^**^	226.41^**^	78.15^**^	51.41^**^	26.43^**^	17.17^**^	ns
Y × IMS	2	8.52^**^	7.74^**^	69.12^**^	22.14^**^	25.91^**^	ns	5.12^**^	5.41^**^
FL × IMS	2	17.28^**^	3.46^*^	36.81^**^	ns	18.35^**^	15.09^**^	8.90^**^	12.70^**^
Y × FL × IMS	2	8.70^**^	ns	ns	ns	17.19^**^	18.25^**^	7.38^**^	11.73^**^

F-values and significance levels (

** *P < 0.01*,

* P < 0.05

*and ns P ≥ 0.05). df, Degree of freedom*.

### Seed weight and seed size

The simultaneous increases in the oil and protein yields can be attributed to the high embryo and seed weights, which compensated for the decreased oil content under IMS_2_ and decreased protein contents under IMS_1_. Thus, the seed development process, including seed weight and size responses to environmental conditions and IMS, was quantified in developing cottonseed.

**Figure 3:**
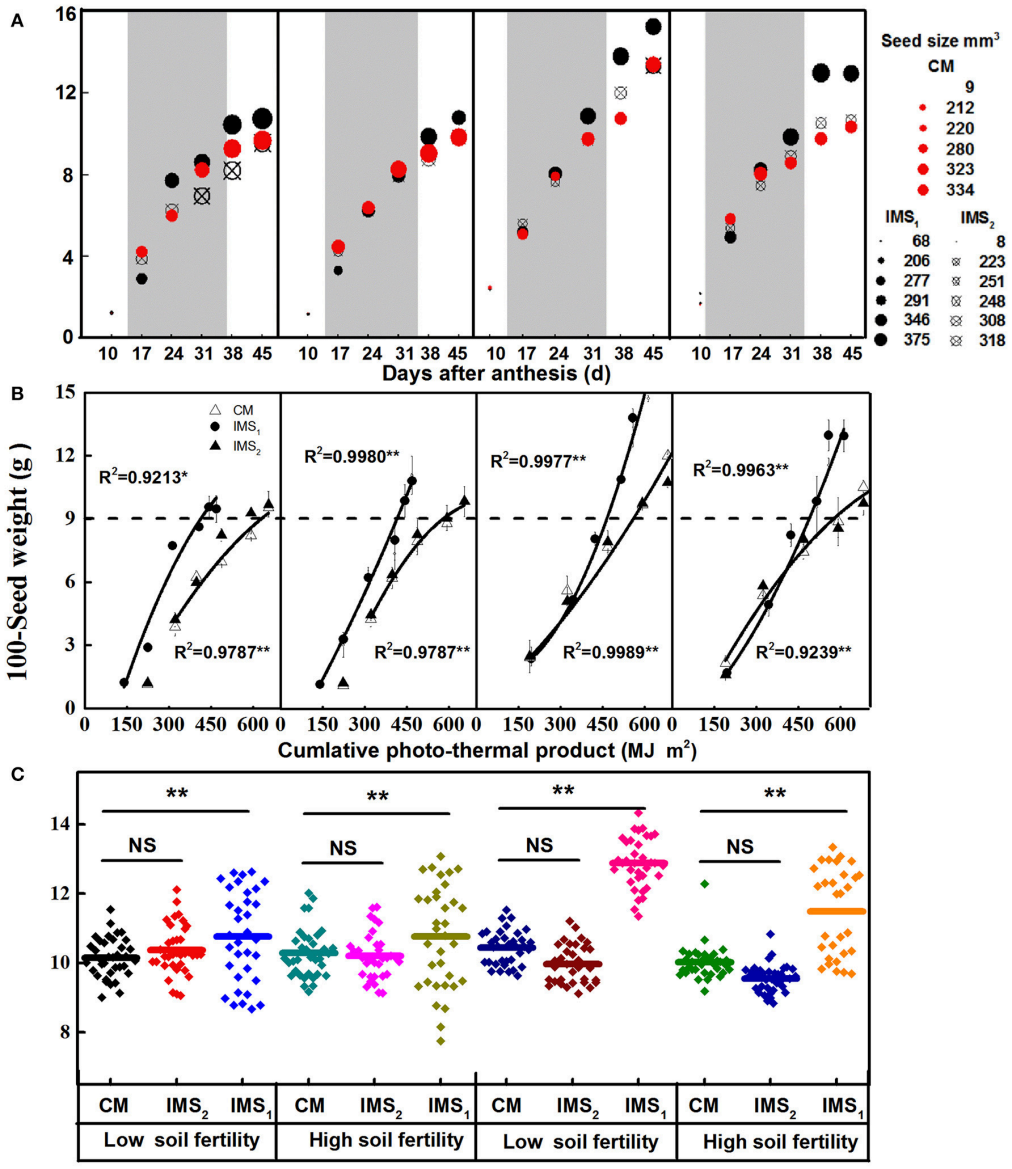
**Seed growth in response to environmental conditions and integrated management strategies. (A)** The seed weights (*y*-value) and sizes (circle size) of cottonseed following anthesis at a fruit position along the 7th sympodial branches. **(B)** The seed weight in response to the cumulative photo-thermal product. The cumulative photo-thermal product represents the combined effect of photosynthetic active radiation (PAR) and air temperature. **(C)** Box-plot comparisons of 100-seed weight distributions throughout a whole plant under three management treatments at two soil fertility levels. The box-plot shows, the median value of the 100-seed weight and the data range of the upper and quartile whiskers in cottonseed (*n* = 24). CM and IMS represent conventional management practice and integrated management strategies, respectively. ^*^ and ^**^ denote significance at the 0.05 and 0.01 levels, respectively.

The developmental profiles for the 100-seed weight and seed size showed typical growth curves (Figure 3A). The seed weight, expressed as grams per 100 seeds, increased dramatically from 17 to 38 DAA. At maturity, the 100-seed weight determined by a random collection of seeds at each fruit position ranged from 7.7 to 13.4 g in 2012 and from 8.8 to 14.7 g in 2013 (Figure 3C). Under IMS_1_, the 100-seed weight at the 7th sympodial branch was higher than that under CM by 7.59 and 15.8% on average at the low and high soil fertility levels, respectively, and was higher in 2013 than in 2012 (Figure 3A). Additionally, no significant differences between IMS_2_ and CM were observed.

The seed size continued to increase from 10 to 24 DAA and remained stable thereafter. The average seed size under CM, IMS_1_ and IMS_2_ was 295, 345, and 289 mm^3^, respectively (Figure 3A), and there were no significant differences in seed sizes between the two soil fertility levels. The seed size was higher under IMS_1_ than under CM, but did not significantly differ between IMS_2_ and CM.

ANOVA revealed that the year (Y), soil fertility level (FL), and the use of integrated management strategies (IMS) had significant effects on the seed weight and size. Their interactions were also significant, except for the Y × FL × IMS interaction, indicating that the seed weight response to FL and IMS depended on the environmental factors of each year. Regarding the photo-thermal requirements of developing cottonseed, the cumulative PTP can reflect the combined effects of solar radiation and air temperature on seed weight accumulation (Figure 3B). The 100-seed weight increased with the PTP during seed growth. The PTP was greater in 2013 than in 2012. During the stage when the seeds weighed up to 9 g, the cumulative PTP was higher under CM than under IMS_1_ and IMS_2_. These results suggest that the increased seed weight observed under the IMS was the result of the combined effect of PAR and air temperature during seed growth and that the seeds under IMS_1_ more efficiently utilized PAR than did those under CM and IMS_2_.

### Embryo biomass accumulation and partitioning into oil and protein

**Figure 4:**
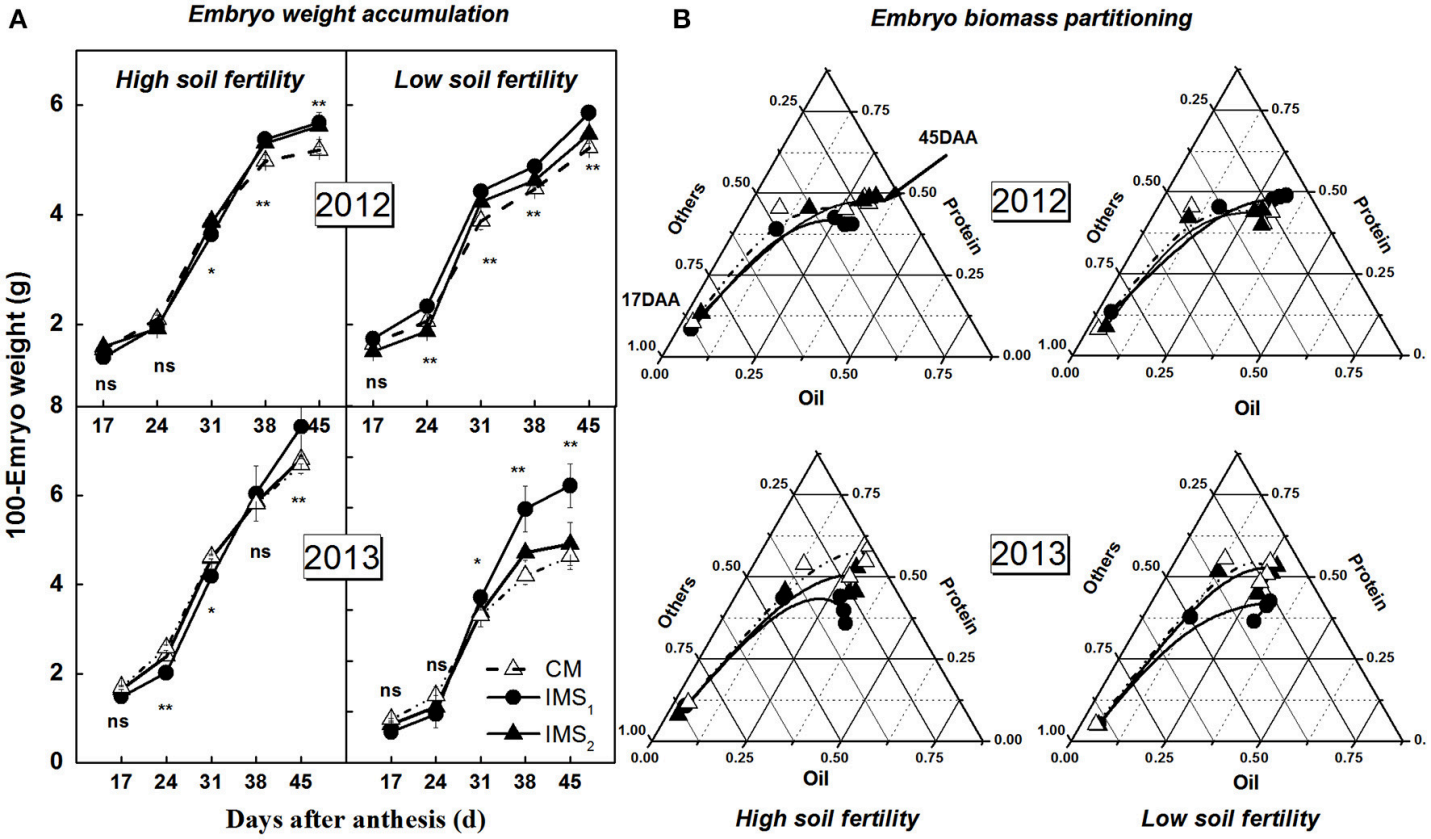
**Embryo weight accumulation (A)** and biomass partitioning into oil and protein **(B)** under conventional management practices and two integrated management strategies at two soil fertility levels in 2012 and 2013. The fraction of oil and protein calculated as the ratio of their dry weight to embryo weight. CM and IMS represent conventional management practice and integrated management strategies, respectively. For each sample point, we tested at least 3 independent samples. The data points are the averages, and the bars = SEs. ^*^ and ^**^ denotes significance at the 0.05 and 0.01 levels, respectively.

The start (DAA_1_) and termination (DAA_2_) times of embryo weight accumulation, the duration of these two time points, and the peak embryo weight accumulation rate, as determined using a sigmoidal growth function, were used to quantify the embryo weight accumulation characteristics (Figure 4A). Rapid embryo weight accumulation was initiated at 15–22 DAA and terminated at 36–44 DAA, lasting for 19–24 days in 2012 and 2013 (Table 4). The peak embryo weight accumulation rate was positively correlated with the embryo weight at maturity. At maturity, the embryo weights under IMS_1_ and IMS_2_ increased by 11.26 and 5.22% at the high soil fertility level and by 19.79 and 4.77% at the low soil fertility level, respectively, compared that under CM. Therefore, the increased embryo weight observed with the IMS was due to the high embryo weight accumulation rate from 16 to 41 DAA.

**Table 4:** **100-Embryo weight accumulation characteristics under conventional management practices and two integrated management strategies at two soil fertility levels in 2012 and 2013**.

**Soil fertility levels**	**Integrated management strategies**	**Embryo weight accumulation characteristics**											
		**DAA**_**1**_ **(days)**		**DAA**_**2**_ **(days)**		**Duration (days)**		**Peak rate (g days**^**−1**^**)**		**100-embryo weight (g)**		***R***^**2**^	
		**2012**	**2013**	**2012**	**2013**	**2012**	**2013**	**2012**	**2013**	**2012**	**2013**	**2012**	**2013**
**(A)**													
High soil fertility	CM	16.6	18.4	35.7	39.2	19.2	20.8	0.197	0.244	5.18b	6.68c	0.9514	0.9793
	IMS_1_	19.1	22.3	38.5	44.8	19.4	22.5	0.221	0.280	5.68a	7.54a	0.9588	0.9843
	IMS_2_	18.1	17.4	38.2	38.6	20.1	21.2	0.210	0.234	5.62a	6.81b	0.9369	0.9859
	Average	17.9	19.3	37.5	40.8	19.6	21.5	0.209	0.252	5.49	7.01		
	CV%	7.24	13.36	4.11	8.39	2.49	4.16	5.81	9.55	4.97	6.61		
**(B)**													
Low soil fertility	CM	15.4	13.2	39.0	37.4	23.6	24.1	0.166	0.156	5.22c	5.05c	0.9396	0.9504
	IMS_1_	17.2	19.6	36.1	38.2	18.9	18.6	0.204	0.258	5.86a	6.43a	0.9005	0.9452
	IMS_2_	15.6	15.9	37.9	37.0	22.3	21.1	0.191	0.187	5.47b	5.29b	0.9348	0.9206
	Average	16.1	16.2	37.7	37.5	21.6	21.3	0.187	0.200	5.52	5.59		
	CV%	6.11	19.61	3.84	1.64	11.14	12.95	10.30	25.87	5.85	13.19		

*The embryo weight accumulation was characterized using the sigma growth function (see Material and Methods). CM and IMS represent conventional management practices and integrated management strategies, respectively. DAA_1_ is the time at which embryo weight accumulation began. DAA_2_ is the time at which embryo weight accumulation ended. The duration is the difference in days between DAA_1_ and DAA_2_. CV% stands for the coefficient of variation. The means within each column followed by the same letter are not significantly different (P < 0.05) according to the least significant difference (LSD) test*.

The partitioning of biomass into oil, protein, and carbohydrate in developing cotton embryos is illustrated in Figure 4B. The proportion of embryo oil and protein increased with embryo growth, with dramatic increases detected from 17 to 24 DAA. At maturity, the embryo oil and protein accounted for approximately 75% of the total embryo dry weight. The embryo oil and protein percentages were higher under IMS_1_ and IMS_2_ than under CM. These results indicate that the IMS not only increase the embryo weight accumulation and final embryo weight but also increase the partitioning of biomass into oil and storage protein during the most active phases of oil and protein biosynthesis.

### Plasma membrane H^+^-ATPase and H^+^-PPase in developing cotton embryos

**Figure 5:**
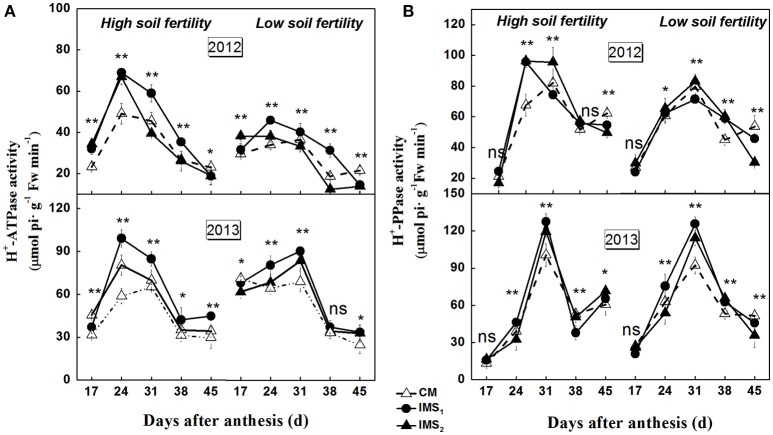
**Developmental profiles of plasma membrane H^**+**^-ATPase (A)** and H^+^-PPase **(B)** activities in developing embryos under conventional management practices and two integrated management strategies at two soil fertility levels in 2012 and 2013. CM and IMS represent conventional management practices and integrated management strategies, respectively. For each sample point, we tested at least 3 independent samples. The data points are the averages, and the bars = SEs. ^*^ and ^**^ denote significance at the 0.05 and 0.01 levels, respectively.

The synthesis of storage oil and protein is highly compartmentalized within embryo cells, and isotope-based methods demonstrate that the biosynthesis of oil and protein use sucrose as their carbon skeleton. H^+^-ATPase and H^+^-PPase tightly regulate proton (H^+^) gradients and mediate the secondary transport of sucrose across the plasma and endosomal membranes. The plasma membrane H^+^-ATPase and H^+^-PPase activities were significantly higher in 2013 than in 2012 (Figure 5). The maximum activities were observed at 24–31 DAA, and were significantly higher under IMS_1_ and IMS_2_ than under CM. The results indicate that the plasma membrane H^+^-ATPase and H^+^-PPase activities were higher during 24–31 DAA than on the last sampling dates. The IMS enhanced the transport of sucrose across the plasma membrane, and the transport of organic solutes into the embryo cells increased under IMS_1_ and IMS_2_ compared with that under CM.

### Sucrose, D-glucose, D-fructose and starch contents in developing cotton embryos

**Figure 6:**
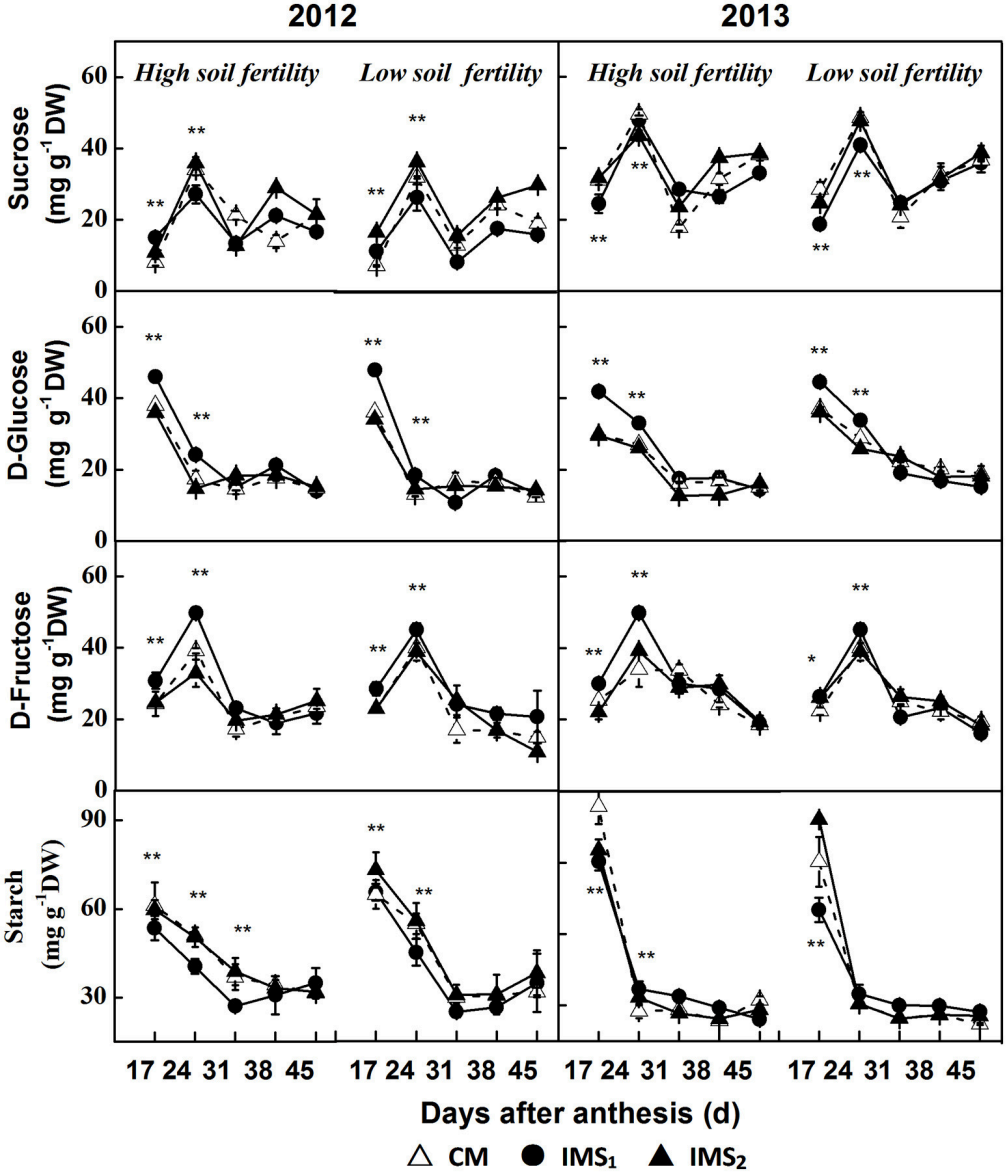
**The sucrose, D-glucose, D-fructose, and starch contents in developing embryos under conventional management practices and two integrated management strategies at two soil fertility levels in 2012 and 2013**. CM and IMS represent conventional management practices and integrated management strategies, respectively. For each sample point, we tested at least 3 independent samples. The data points are the averages, and the bars = SEs. ^*^ and ^**^ denote significance at the 0.05 and 0.01 levels, respectively.

The sucrose content peaked at 24 DAA and slightly increased after 31 DAA. Significant differences in the peak sucrose content were detected (Figure 6), and the compartments of each treatment showed that sucrose content was significantly lower under IMS_1_ than under IMS_2_, and CM. However, no significant difference was observed between IMS_2_ and CM. The sucrose content decreased by 11.8 and 16.7% on average at high and low soil fertility levels, respectively.

The D-glucose content decreased over time during the seed growth period (Figure 6). Significant differences were observed before 31 DAA in 2012 and 2013. The D-glucose contents for IMS_1_ were significantly higher than those under CM at high and low soil fertility levels. However, no significant differences were observed between CM and IMS_2_. The D-glucose content increased by 30.6 and 28.4% on average at the high and low soil fertility levels, respectively, over the 2 years.

The D-fructose content peaked at 24 DAA and decreased with seed growth (Figure 6). At both high and low soil fertility levels, the D-fructose content under IMS_1_ was significantly higher than that under CM before 31 DAA. The D-fructose content increased by 19.6 and 16.9% on average at both high and low soil fertility levels, respectively.

The starch content was higher at 17 and 24 DAA and remained relatively stable thereafter (Figure 6). The starch content obviously decreased when IMS were used and the starch content in the samples collected in 2013 was higher than that collected in the samples collected in 2012. Compared with that under CM, the starch content under IMS_1_ and IMS_2_ decreased by 12.4 and 16.9% at the high and low soil fertility levels, respectively. By analyzing the development profiles of sucrose and the D-glucose, D-fructose and starch contents, the IMS were found to increase the D-glucose and D-fructose contents but decrease the sucrose and starch contents. The utilization of hexoses for starch biosynthesis was transient during the early stage of cotton embryo growth.

### Activities of sucrose metabolic enzymes in developing cotton embryos

**Figure 7:**
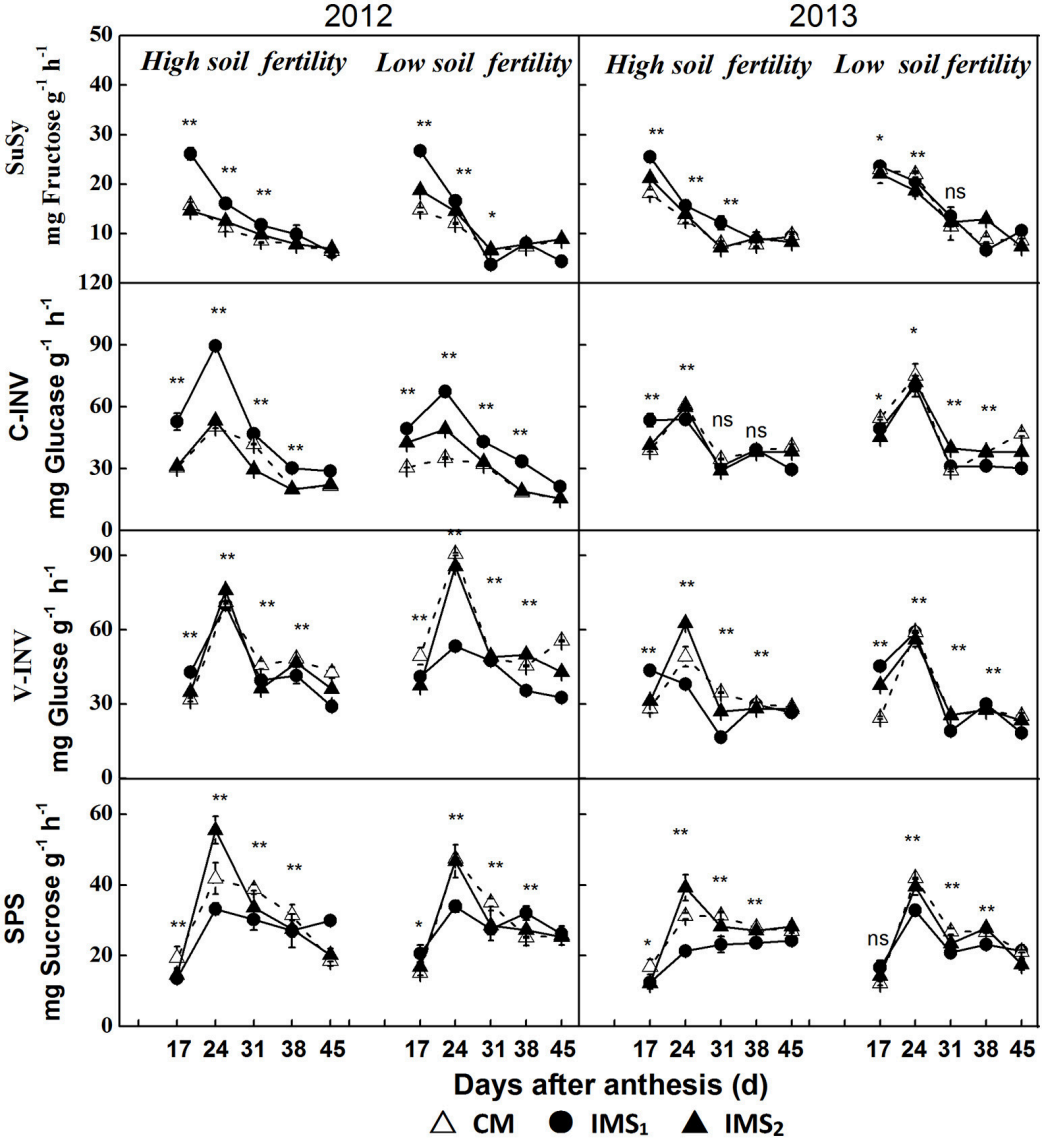
**Enzymes involved in the sucrose metabolic pathway in developing embryos**. CM and IMS represent conventional management practices and integrated management strategies, respectively. Sucrose synthase (SuSy), cell wall invertase (C-INV), vacuolar invertase (V-INV), and sucrose phosphate synthase (SPS) are illustrated. For each sample point, we tested at least 3 independent samples. The data points are the averages, and the bars = SEs. ^*^ and ^**^ denote significance at the 0.05 and 0.01 levels, respectively.

Enhanced sucrose/H^+^ symport increased the D-glucose and D-fructose contents that resulted from the high cleavage rate of sucrose rather than from the actual sucrose content. Therefore, the cleavage of sucrose into hexoses by invertase (INV) and sucrose synthase (SuSy) and the synthesis of sucrose by sucrose phosphate synthase (SPS) were quantified using frozen embryos at various stages after anthesis (Figure 7).

The samples collected at 17 DAA had the highest SuSy activities, which decreased over time after anthesis (Figure 7). The SuSy activity was significantly higher under IMS_1_ than under IMS_2_ and CM before 31 DAA. However, no significant differences between IMS_2_ and CM were observed. The SuSy activity increased by 37.0 and 11.3% on average at the low and high soil fertility levels, respectively.

During seed growth, significant differences in the activities of C-INV (located in the cell wall) and V-INV (located in the vacuole) were observed in developing cotton embryos (Figure 7). IMS_1_ resulted in increased C-INV activity and decreased V-INV activity compared with IMS_2_ and CM at the various growth stages, indicating that the hydrolysis rate of sucrose into hexoses was higher in the plasma membrane than in the vacuolar membrane.

The SPS activity peaked at 31 DAA in 2012 and 2013 (Figure 7). In contrast to SuSy activity, SPS activity was significantly lower under IMS_1_ than under CM. The effects of the IMS on SPS activity were significant at 24–31 DAA. The SPS activity decreased by 37.3 and 12.4% on average at low and high soil fertility levels, respectively.

An assessment of the carbohydrate profiles along with the enzyme activities involved in sucrose metabolism further demonstrated that the increased hexoses contents that under the IMS were attributed to the enhanced cleavage rate of sucrose and the low level of sucrose biosynthesis in developing cotton embryos.

### Bayesian inference of network-based mutual relationships

**Figure 8:**
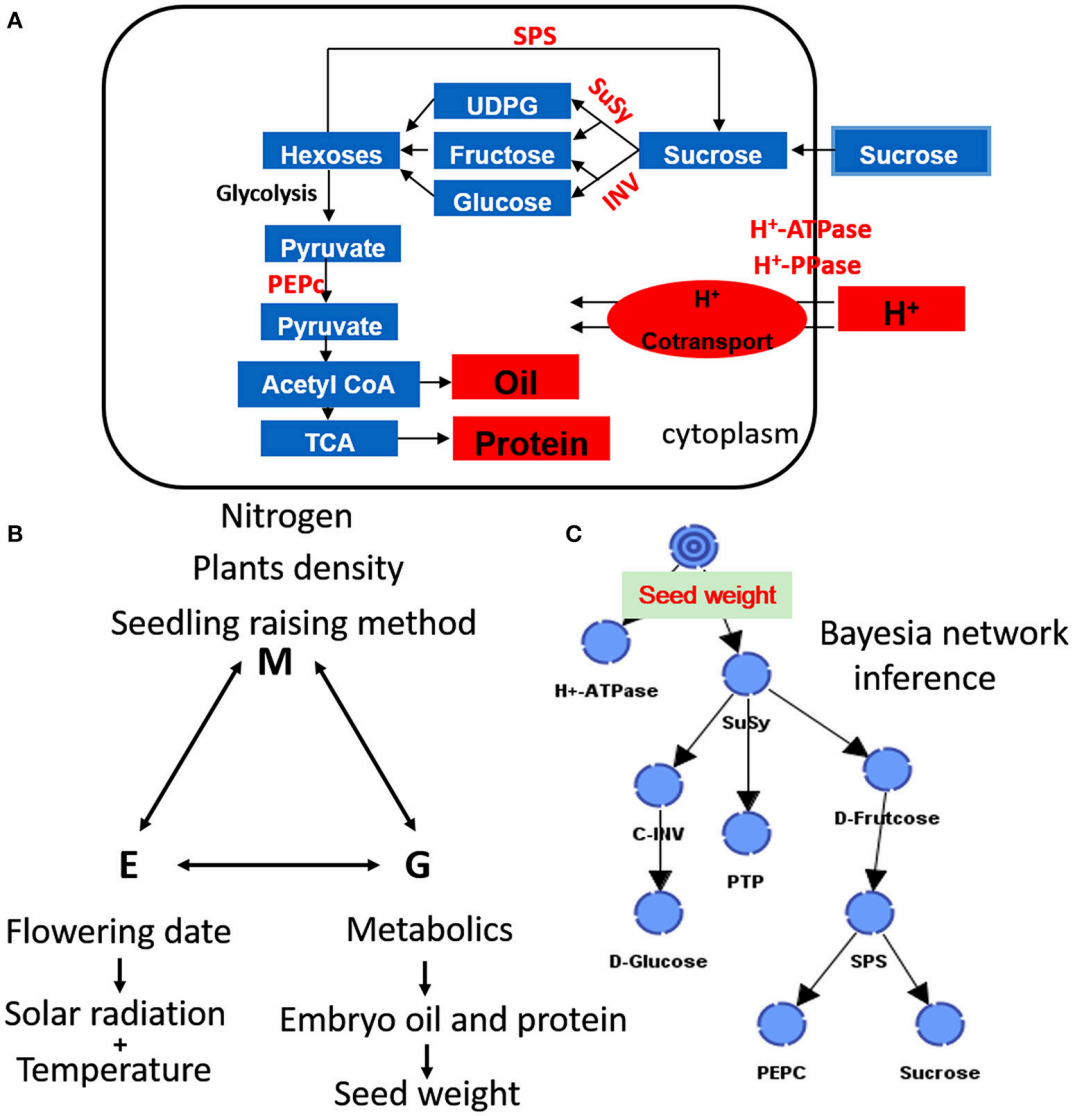
**A comprehensive and simplified scheme of seed growth, biosynthesis of storage oil and protein (A)**, and their interactions with the environment and management strategies **(B,C)** in developing cottonseed.

**Figure 9:**
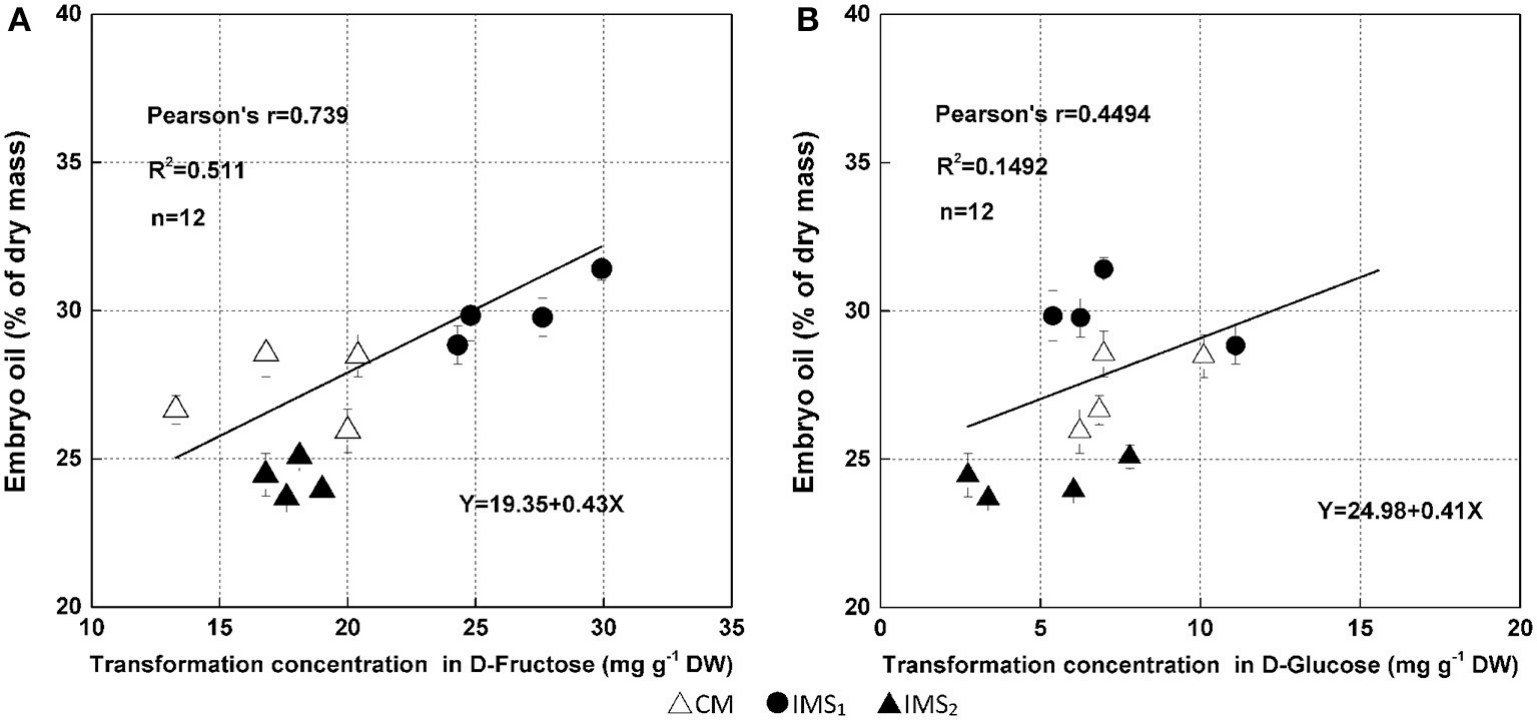
**Relationships between embryo oil and hexoses (D-fructose, A; D-glucose, B) contents**. Changes in hexoses contents were calculated using the embryo samples that were collected at 38 DAA excluding those collected at 17 DAA. The lines indicate linear regression lines.

The results obtained from the Bayesian network analysis provided insights into the correlation between the 100-seed weight and carbohydrate metabolic levels (Table 5, Figures 8A–C). Inferences were based on the calculation of probabilities during seed growth (*n* = 96). The 100-seed weight was set as the target node, and the key metabolic events were expected to be directly responsible for the variations in seed weight. Notably, the contributions of SuSy activity and D-fructose content to the variations in seed weight reached 47.02 and 29.05%, respectively (Table 5). Additionally, the embryo oil content was positively correlated with the hexoses contents; the slope of the fitted line for D-fructose was higher than that for D-glucose (Figure 9). These results indicate that IMS increased the rate of sucrose hydrolysis, thereby increasing the hexoses contents and contributing to the increased seed weight. SuSy and D-fructose play dominant roles in seed weight accumulation.

**Table 5:** **Node significance with respect to the information gain contributed by the node to the known 100-seed weight**.

**Node**	**Normalized mutual information (%)**	**Relative significance**	***G*****-test**
SuSy	47.02	1	98.039
D-Fructose	29.05	0.618	60.584
C-INV	19.64	0.418	40.948
SPS	11.73	0.249	24.461
D-Glucose	10.17	0.216	21.206
PEPC	7.54	0.160	15.723
H^+^-ATPase	7.19	0.153	14.995
Sucrose	3.56	0.076	7.427

*The 100-seed weight was set as the target node. The relationships were inferred using the algorithm for the Bayesian network*.

## Discussion

This study showed that management of a high-yielding cultivar (Siza-3) management with an economic N rate (375 kg ha^−1^), an optimal N application schedule, adequate plant density (30,000 plant ha^−1^), and use the substrate seedling raising method can increase cottonseed, oil, and protein yields (Table 1). IMS_1_ produced the highest oil yield and IMS_2_ produced the highest protein yield at both high and low soil fertility levels. Fiber yield also increased markedly (*P* < 0.01). The use of an economic N rate and optimal N application schedule (Chen et al., 2016) increased the soil available N content, which supplied enough N at the flowering and boll-forming stages, thereby increasing protein content in cotton embryos (Yang et al., 2016). The optimal plant density prevented over-shading and increased the cumulative light interception, thereby increasing cottonseed yield (data not shown). Therefore, methods that optimize the canopy architecture and economically increase the soil available N are beneficial to cottonseed, oil and protein yields.

Soil fertility, also referred to soil quality, is known as “providing yields” (Patzel et al., 2000) and varies across farmlands (Nafziger, 2012). Small farm holders should consider soil fertility when designing crop management schemes to improve resource use efficiency (Tittonell et al., 2007; Zingore et al., 2007). Previous studies have classified soil fertility levels based on nutrition status, especially the contents of soil organic matter (SOM), and the total nitrogen (TN) contents (Tiessen et al., 1994; Mtambanengwe and Mapfumo, 2005). In this study, the yield of cotton grown in soils with high SOM (17.76 ± 0.69 g kg^−1^) and TN (0.84 ± 0.03 g kg^−1^) contents was significantly higher than that of cotton grown in soil with low SOM (14.31 ± 0.62 g kg^−1^) and TN (0.76 ± 0.04 g kg^−1^) contents. Therefore, the soil with high SOM and TN contents were classified as high-fertility soil, whereas soils with low SOM and TN contents were classified as low-fertility soil. Comparisons of the yield performance between the two soil fertility levels suggest that soils with high SOM and TN contents play a dominate role in increasing cottonseed, oil, and protein yields. ANOVA (Table 2) suggested that the environmental conditions and soil nutrition status were the primary factors causing yield fluctuation between the 2 years. Therefore, the design of management strategies should consider both environmental conditions and soil nutrition status, especially the photo-thermal environment during seed growth, and soil organic matter and total nitrogen contents.

The variations in seed weight and seed size between the 2 years were non-negligible in significance for the stable yields of cotton plants (Figure 3) not only because the seed weight is a component of cottonseed yield but also because seed weight is correlated with fiber elongation and seed reserve accumulation (Buchala, 1987; Ruan et al., 2000). Variations in seed weight and seed size in the same genotype result from environmental conditions, crop management practices, and their combined effects (Etterson and Galloway, 2002; Spurr et al., 2002). Paternal and maternal light environments influence the offspring seed mass (Etterson and Galloway, 2002). Seed growth also self-adjusts to adapt to variations in environmental conditions, such as changes in the flowering date and boll age (Li et al., 2009). The PTP, which can reflect the combined effects of solar radiation and temperature, is a driving force for seed growth (Li et al., 2009). In this study, the results (Table 2) suggest that seed weight responses to FL and IMS depend on environmental factors. The 100-seed weight and seed size were significantly higher in 2013 than in 2012, which could be explained by the relatively higher PTP during boll development in 2013 (613–760 MJ m ^−2^). In addition, the increased PTP decreased boll age by 3–6 days in 2013. Using IMS_1_ as an example, from flowering to 45 DAA, the PTP was 30.79% higher in 2013 than in 2012, and the boll ages in 2012 and 2013 were 54 and 48 days, respectively. This indicates that the key factor for the increased yield in 2013 relative to that in 2012 was the increased PTP during seed development, which resulted in the high seed weight in 2013. However, in this study, the seed weight responses to environmental conditions and IMS during various stages of seed development were different; thus, a critical stage existed.

Heavier and larger seeds have great potential to amass higher oil and protein contents (Önder and Babaoglu, 2001). The N rate increases the seed weight (Sawan et al., 2009). In this study, the 100-seed weight and seed size were significantly higher under IMS_1_ than under IMS_2_ and CM, whereas no significant differences were observed those value under IMS_2_ and CM. The seed size continued to increase before 24 DAA and remained stable thereafter, which is consistent with a previous report (Stewart, 1986). Due to the difference in boll-forming stages (data not shown) that resulted from different seedling-raising methods, the PTP under IMS_2_ and CM was higher than that under IMS_1_. However, when the seeds weighed 9 g, the PTP was lower under IMS_1_ than that under CM and IMS_2_. These results suggest that IMS_1_ is more productive and efficient than CM and IMS_2_ in utilizing PTP.

The higher peak embryo weight accumulation rate and prolonged accumulation duration resulted increase the embryo weight (Figure 4, Table 4), indicating that the final embryo weight was attributed to the duration and maximum rate of dry matter accumulation, which is consistent with a previous report (Tang and Xiao, 2013). Similar studies have also investigated boll weight formation (Liu et al., 2015) and cellulose accumulation (Wang et al., 2009), suggesting that the final boll weight and fiber strength are controlled by the maximum accumulation rate and their duration during the boll-forming stage. Interestingly, we also observed increased partitioning of biomass into oil and protein under IMS_1_ and IMS_2_ compared that under CM. These findings are attributed to the increased hexoses content in the embryos because hexoses contribute to the increment of oil and protein contents (Figure 9).

The increased cottonseed yield was accompanied by alterations in the storage researves compositions within cottonseed embryos (Figure 2). Increasing the N supply increases the seed protein content without affecting the seed oil content (Rotundo and Westgate, 2009; Zanetti et al., 2009; Mayer et al., 2012). However, in sunflower seeds, the oil, and protein contents increased in response to increased N supply (Jalilian et al., 2012). In this study, increasing the cottonseed yield per 100 kg was accompanied by a 0.53% increase in protein content, but a 0.45% decrease in oil content, which was consistent with previous reports in soybean (Wilcox and Cavins, 1995; Specht et al., 1999; Bellaloui et al., 2015), maize (Duvick and Cassman, 1999) and cottonseed (Sawan et al., 2001; Main et al., 2013; Pettigrew and Dowd, 2014). The alterations in seed storage reserves contents are due to eight quantitative trait loci (QTLs) that are co-localized in the same regions but have opposite additive effects (Yu et al., 2012). Although the IMS altered the embryo oil and storage protein compositions by increasing the N dose and N application schedule, the high embryo weight accumulation rate and high proportion of biomass partitioning into oil and storage protein compensated for the reduced oil content under IMS_2_ and the reduced protein content under IMS_1_. These results reveal that embryo weight contributes the most to the final oil and protein yields. Producers can use different management practices to produce cotton embryos with a high protein or oil content.

The high oil content produced under IMS_1_ and the high protein content produced under IMS_2_ were advantageous for elucidating the physiological basis of high-yielding cotton embryos. In this study, it was hypothesized that the carbon supply is an important factor that contributes to the high productivity of cottonseed. Evidence related to sucrose/H^+^ symport, sucrose hydrolysis, and hexoses contents were important for test this hypothesis.

Co-suppression of the H^+^-ATPase isoform demonstrated that the H^+^-ATPase creates a proton gradient across the plasma membrane to promote sucrose/H^+^ transport (Zhao et al., 2000). Overexpressing the H^+^-PPase gene increases sucrose degradation and starch synthesis by regulating the enzymes involved in sucrose degradation (Geigenberger et al., 1998; Wang et al., 2014). In this study, the increased H^+^-ATPase and H^+^-PPase activities indicated that sucrose/H^+^ symport was more active in the IMS_1_ and IMS_2_ embryos (Figure 5). Interestingly, the D-fructose and D-glucose contents were significantly higher and the sucrose and starch contents were significantly lower under IMS_1_ than for IMS_2_ and CM (Figure 6), which is inconsistent with previous findings (Rosche et al., 2002). This discrepancy was due to the high cleavage rate of sucrose in developing cotton embryos. The evaluation of the enzymatic activities involved in sucrose metabolism demonstrated that the SuSy and C-INV activities were significantly increased, whereas those of SPS and V-INV significantly decreased under IMS_1_ compared with those under IMS_2_ and CM (Figure 7). Hexoses are correlated positively with the SuSy and C-INV activities and negatively with activities of SPS and V-INV. Similar results have also been reported in maize (Miller and Chourey, 1992), tomato (Jin et al., 2009), and sugarcane (Verma et al., 2011), in which the enzymes responsible for the cleavage of sucrose into hexoses regulate sink strength, sucrose-hexoses balance, and seed development (Weber et al., 2005; Ruan et al., 2008). These previous studies suggested that sucrose/H^+^ symport increases the amount of sucrose imported into embryos and that the high cleavage rate of sucrose contributes to the high D-fructose and D-glucose levels. As the embryos grew, the activities of SuSy, and C-INV were high and then decreased regularly over time, synthesizing large amounts of hexoses via sucrose metabolism during the early stage of embryo growth, resulting in rapid oil and protein accumulation in a short period of time (from 24 to 38 DAA), controlling over 70% of the embryo oil production. Furthermore, the decreasing hexoses contents correspond to rapid oil accumulation and they were significantly and positively correlated with the final embryo oil content (Figure 9). The most beneficial effect of the high hexoses contents in the early stage of embryo growth was the increased carbon sources that could serve as a carbon skeleton for embryo oil and protein biosynthesis.

These results indicate that SuSy plays a critical role in early embryo development by regulating sucrose hydrolysis and embryo weight accumulation (Figure 8C, Table 5), which is consistent with the results of previous studies (Ruan et al., 1997). However, the critical stage was not the most active phase of oil and protein accumulation, but rather the early stage of embryo growth, which was highly convoluted. Seed development associated with the uptake of photosynthates from subtending leaves, but the photosynthetic capacity of subtending leaves decreases over time (Oosterhuis, 2001). Additionally, the biosynthesis of hexoses into oil and storage protein is a process that consumes a considerable amount of energy and results in the accumulation of 70% of the total oil and protein during the maturation stage (24–38 DAA). It has been suggested that embryos have a pre-storage step for oil and protein biosynthesis, as characterized by the rapid cleavage of sucrose and the accumulation of large amount of hexoses prior to the biosynthesis of oil and protein. Similar studies on seed of *Vicia faba* L have also reported this result, suggesting that a high hexoses contents not only contributes to oil and storage protein contents but also regulates cell division and seed development (Weber et al., 1996). Interestingly, mature cotton embryos have two large cotyledons that accumulate lipids and storage protein but not starch (Liu et al., 2012). In this study, the use of hexoses for starch biosynthesis was transient during the early stage of cotton embryo growth (Figure 6). Starch acted as the transitional sink that accumulated during the early stage of embryo growth and degraded into hexoses for oil and storage protein biosynthesis during the maturation stage (24–38 DAA).

The results presented in this study support our hypothesis that carbohydrate metabolism is an important factor that controls the productivity of cotton oil and storage protein. However, optimal N management increases the cumulative intercepted PAR, radiation use efficiency, and water use efficiency, thereby increasing cottonseed yield (Ahmad et al., 2015). An optimal plant density increased the cumulative light interception, which in turn increased cottonseed yield (Sawan et al., 1993, 2001; Xue et al., 2015). Further research may explore the canopy radiation interception and nitrogen use efficiency to determine the systematic advantages of IMS for cotton production. Furthermore, whether IMS can be used for the management of other cotton production regions requires further study based on analyses of the factors that limit crop productivity and quality under a given set of environmental conditions.

## Conclusions

Over the 2-year study, an economic N rate, optimal N application schedule, adequate plant density, and use of the seedling-raising method integrated as a coherent management system resulted in an increased cottonseed, oil and protein yields. Although the IMS altered the embryo oil and protein contents, the increased embryo weight compensated for the reduced oil content under IMS_2_ and for the reduced protein content under IMS_1_. Therefore, IMS_1_ and IMS_2_ showed significant advantages over CM in terms of cottonseed, oil, and protein yields at high and low soil fertility levels. Furthermore, the IMS regulated the flowering date and cumulative PTP during seed growth, thereby increasing seed weight. In developing cottonseed, the IMS increased the embryo weight accumulation rate and biomass partitioning into oil and protein, which was attributed to increases in sucrose/H^+^ symport, sucrose hydrolysis, and hexoses rather than the sucrose and starch contents. The enhanced SuSy activity and D-fructose content under IMS played predominant roles in seed development and embryo oil and protein biosynthesis. These findings of the study suggest that the following cotton management strategies should be considered: (1) A high-yielding and ecologically adapted cultivar should be planted; (2) seedlings should be raised with bio-organic fertilizer to produce stronger seedlings; (3) a high plant density should be used for high-yielding plant populations; (4) an economic N fertilization rate should be used, with applications when needed by the plants (flowering and boll-forming stages); and (5) the design of management strategies should consider both photo-thermal environment and soil nutrition status. These management practices are highly recommended for cotton management integrating local optimal management practices and hence maximize the yields of cottonseed, oil and protein.

## Author contributions

ZZ, YM, and BC conceived the idea and led the study design. HY performed the experiment and analysis and wrote the paper. XZ assisted in plant sampling and laboratory analysis. WZ and YW assisted in manuscript writing and editing.

## Funding

This study was financially supported by the Natural Science Foundation of China (31371583 and 31401327), China Agriculture Research System (CARS-18-20) and the Special Fund of National Public Welfare Industry (Agriculture) R & D Program (201203096 and 201303002).

### Conflict of interest statement

The authors declare that the research was conducted in the absence of any commercial or financial relationships that could be construed as a potential conflict of interest.

## Abbreviations

CM: conventional management practices
IMS: integrated management strategies
HF: high soil fertility
LF: low soil fertility
DAA: days after anthesis
PAR: photosynthetic active radiation
PTP: cumulative photo-thermal product
H^+^-PPase: inorganic pyrophosphatase
SuSy: sucrose synthase
C-INV: cell wall invertase
V-INV: vacuolar invertase
SPS: sucrose phosphate synthase.
